# Supplementary material for: Convergent and Divergent Connectivity Patterns of the Arcuate Fasciculus in Macaques and Humans
Source: Adv Sci (Weinh). 2025 Dec 15;13(13):e14352. doi: 10.1002/advs.202514352 (PMC12955879; doi:10.1002/advs.202514352)
Supplement: Supplementary file 1 — Supporting Information [file ADVS-13-e14352-s002.docx]

**Supporting Information**

**Convergent and divergent connectivity patterns of the arcuate fasciculus in macaques and humans**

Jiahao Huang, Ruifeng Li, Wenwen Yu, Anan Li, Xiangning Li, Mingchao Yan, Lei Xie, Qingrun Zeng, Xueyan Jia, Shuxin Wang, Ronghui Ju, Feng Chen, Qingming Luo, Hui Gong, Andrew Zalesky, Xiaoquan Yang*, Yuanjing Feng*, and Zheng Wang*

**Fluorescent signal distribution:** Four macaque monkeys were used in this study (Table.S7), and all subjects underwent ex-vivo 11.7T dMRI scanning and fMOST imaging. To register the original images of fMOST (0.65 × 0.65 × 3.00μm) with the Cyno162 macaque atlas, we utilized the deformation field and transformation matrix derived from the registration of down-sampled fMOST images (300 × 300 × 300μm). In a common space, we performed fluorescence signal extraction and statistical analysis on the fMOST images of the macaque brain. For the extraction of fluorescence signal distribution from fMOST images with specific steps as follows: First, we registered the cortical parcellation to the space of down sampled fMOST coronal slice images (300μm × 300μm × 300μm). Then, we applied the modified maximum entropy threshold segmentation algorithm to the registered coronal images to segment fluorescent fiber signals from the background. To ensure segmentation accuracy, we manually checked and corrected the segmentation results. Next, we calculated the signal density of binary images in each brain region of interest. For each segmented image, signal density was calculated as follows: the sum of detected pixels in each brain region of interest divided by the sum of all brain fluorescent signal pixels. Subsequently, the signal density matrix was used for further quantitative analysis in Amira software (v6.1.1, FEI). The statistical results of fluorescence signals from different divisions of the macaque brain were shown in Table S1-S6.

The experimental details were described in Table S7, including injection cases, viral vectors, viral time, the resolution and data size of fMOST imaging.

**Supplementary Table S1 Projection signals and proportions of frontal lobe.**

| **Acronym** | **Full name of brain areas** | **Signal Volume (μm³)** | **Signal Ratio** |
| --- | --- | --- | --- |
| 12l | lOFC, area 12l, lateral part | 603040248 | 45.89% |
| F5_(6Va/6Vb) | agranular frontal area F5, PMCv | 95266800 | 7.25% |
| 44 | vlPFC, area 44 | 45132984 | 3.43% |
| 45a | vlPFC, area 45a | 16793136 | 1.28% |
| 12o | lOFC, area 12o, orbital part | 15255432 | 1.16% |
| F1_(4) | agranular frontal area F1, M1,4 | 15224328 | 1.16% |
| F2_(6DR/6DC) | agranular frontal area F2, PMdc | 5756832 | 0.44% |
| 46v | dlPFC, area 46v, clPFC | 5322456 | 0.41% |
| PrCO | precentral opercular area, PFC | 5304744 | 0.40% |
| 13m | mOFC, area 13m | 5108400 | 0.39% |
| 12m | lOFC, area 12m, medial part | 4151304 | 0.32% |
| 46d | dlPFC, area 46d, clPFC? | 3519072 | 0.27% |
| 13l | mOFC, area 13l | 3457512 | 0.26% |
| F4_(4C/6Va/6Vb) | agranular frontal area F4, PMCv | 3096144 | 0.24% |
| 9d | dlPFC, area 9, dorsal subdivision | 3014712 | 0.23% |
| F3_(SMA) | agranular frontal area F3, SMA | 2922480 | 0.22% |
| 32 | ACC | 2261088 | 0.17% |
| 10mr | frontal polar antrior part (area 10m) | 2207520 | 0.17% |
| 10mc | frontal polar posterior part (area 10m), mPFC | 1873584 | 0.14% |
| 13b | mOFC, area 13b | 1746792 | 0.13% |
| 8Bs | area 8B in the arcuate sulcus (“s” stands for sulcus) | 1731672 | 0.13% |
| 24b | ACC, area 24b | 1696032 | 0.13% |
| 46f | dlPFC, area 46f, clPFC? | 1648080 | 0.13% |
| F6_(PreSMA) | agranular frontal area F6, PreSMA | 1641816 | 0.12% |
| 14c | mOFC, area 14c | 1440288 | 0.11% |
| 10o | frontal polar antrior part (area 10o) | 1387584 | 0.11% |
| 25 | subgenual ACC, area 25 | 1384344 | 0.11% |
| 45b | vlPFC, area 45b | 1315224 | 0.10% |
| 9m | mPFC, area 9, medial subdivision | 1262952 | 0.10% |
| 8Ad | clPFC, area 8A | 1185408 | 0.09% |
| 8Bm | area 8B, medial subdivision | 1099008 | 0.08% |
| 14r | mOFC, area 14r | 1060992 | 0.08% |
| 11l | mOFC (area 11l) | 1055808 | 0.08% |
| 11m | mOFC (area 11m) | 1003104 | 0.08% |
| 8Bd | area 8B, dorsal subdivision | 953640 | 0.07% |
| F7_(6DR) | agranular frontal area F7, PMdr | 651672 | 0.05% |
| AONl | anterior olfactory nucleus, lateral division | 602208 | 0.05% |
| 12r | lOFC, area 12r, rostal part? | 471096 | 0.04% |
| 13a | mOFC, area 13a | 279936 | 0.02% |
| AONd/m | anterior olfactory nucleus, dorsal/medial division | 152280 | 0.01% |
| 8Av | clPFC, area 8A | 117288 | 0.01% |
| TTv | ventral tenia tectum | 108864 | 0.01% |
| 24a | ACC, area 24a | 107136 | 0.01% |

**Supplementary Table S2 Projection signals and proportions of temporal lobe.**

| **Acronym** | **Full name of brain areas** | **Signal Volume (μm³)** | **Signal Ratio** |
| --- | --- | --- | --- |
| TEO | area TEO | 27387720 | 2.08% |
| TEpd | dorsal subregion of posterior TE | 25560576 | 1.95% |
| TAa | area TAa (sts dorsal bank) | 19308024 | 1.47% |
| TEm | TE 1–5, 74–87, 125 | 14146272 | 1.08% |
| TPO | area TEO | 10973016 | 0.84% |
| STGr | rostral superior temporal gyrus | 10412496 | 0.79% |
| TEav | ventral subregion of anterior TE | 10111824 | 0.77% |
| TEad | dorsal subregion of anterior TE | 10102104 | 0.77% |
| AL | anterior lateral, belt region of the auditory cortex | 8245152 | 0.63% |
| TGa | agranular part of the temporal pole | 7815096 | 0.59% |
| CPB | superior temporal area | 7393248 | 0.56% |
| RPB | superior temporal area, RPB | 7278120 | 0.55% |
| Tpt | TEm | 6897528 | 0.52% |
| 36r | area 36 of the perirhinal cortex, rostral subregion | 5556384 | 0.42% |
| EC | entorhinal cortex, caudal division | 4880952 | 0.37% |
| TEpv | ventral subregion of posterior TE | 4803840 | 0.37% |
| ER | entorhinal cortex, rostral division | 4453272 | 0.34% |
| 36c | area 36 of the perirhinal cortex, caudal subregion | 4369248 | 0.33% |
| TGvd | ventral dysgranular part of the temporal pole | 3870288 | 0.29% |
| TGvg | ventral granular part of the temporal pole | 3641760 | 0.28% |
| TGsts | sts part of the temporal pole | 3452112 | 0.26% |
| 35 | area 35 of the perirhinal cortex | 3446928 | 0.26% |
| TF | area TF of the parahippocampal cortex | 3142584 | 0.24% |
| RTL | lateral rostrotemporal, belt region of the auditory cortex | 3117744 | 0.24% |
| TGdd | dysgranular part of the dorsal temporal pole | 2899584 | 0.22% |
| EI | entorhinal cortex, intermediate division | 2866752 | 0.22% |
| IPa | area IPa (sts fundus) | 2602800 | 0.20% |
| TEa | area TEa (sts ventral bank) | 2351160 | 0.18% |
| 36p | area 36 of the perirhinal cortex, temporal-polar subregion | 2347272 | 0.18% |
| R | rostral, core region of the auditory cortex | 2126520 | 0.16% |
| TFO | area TFO of the parahippocampal cortex | 1540728 | 0.12% |
| ELr | entorhinal cortex, lateral division (rostral part) | 1362096 | 0.10% |
| CL | caudal lateral, belt region of the auditory cortex | 1302264 | 0.10% |
| RTp | rostrotemporal (“p” refers to polar) | 1238544 | 0.09% |
| ECL | entorhinal cortex, caudal limiting division | 1215864 | 0.09% |
| ML | middle lateral, belt region of the auditory cortex | 1134648 | 0.09% |
| TGdg | granular part of the dorsal temporal pole | 1133136 | 0.09% |
| EO | entorhinal cortex, olfactory division | 1007424 | 0.08% |
| PGa | superior temporal area, area Pga | 998784 | 0.08% |
| RT | rostrotemporal, core region of the auditory cortex | 704376 | 0.05% |
| A1 | AI: auditory area I, core region of the auditory cortex | 622080 | 0.05% |
| RM | rostromedial, belt region of the auditory cortex | 514512 | 0.04% |
| ELc | entorhinal cortex, lateral division (caudal part) | 493776 | 0.04% |
| MST | medial superior temporal area | 457056 | 0.03% |
| MT | middle temporal area | 249912 | 0.02% |
| RTM | medial rostrotemporal, belt region of the auditory cortex | 153144 | 0.01% |
| FST | floor of superior temporal area | 104976 | 0.01% |
| CM | caudomedial, belt region of the auditory cortex | 79272 | 0.01% |
| TH | area TH of the parahippocampal cortex | 70416 | 0.01% |

**Supplementary Table S3 Projection signals and proportions of parietal lobe.**

| **Acronym** | **Full name of brain areas** | **Signal Volume (μm³)** | **Signal Ratio** |
| --- | --- | --- | --- |
| 1-2 | somatosensory areas 1 and 2 | 20555856 | 1.56% |
| MIP | medial intraparietal area | 12522384 | 0.95% |
| SII | secondary somatosensory area (S2) | 8104968 | 0.62% |
| 7a_(Opt/PG) | visual area 7a (parietal area Opt/PG) | 5858568 | 0.45% |
| 3a/b | somatosensory areas 3a and 3b | 4415688 | 0.34% |
| 5_(PEc) | somatosensory area 5, PEc | 3882600 | 0.30% |
| 5_(PEa) | somatosensory area 5, PEa | 2729592 | 0.21% |
| 7op | area 7op (parietal operculum) | 2633688 | 0.20% |
| 7m (PGm) | area 7m in the medial parietal cortex (area PG) | 2292408 | 0.17% |
| 7b_(PFG/PF) | visual area 7b (parietal area, PFG/PF) | 1743768 | 0.13% |
| LIPd | lateral intraparietal area, dorsal subdivision | 1372032 | 0.10% |
| VIP | ventral intraparietal area | 933984 | 0.07% |
| 5_(PE) | somatosensory area 5, PE | 811728 | 0.06% |
| LIPv | lateral intraparietal area, ventral subdivision | 604800 | 0.05% |
| AIP | anterior intraparietal area | 333936 | 0.03% |
| LOP | lateral occipital parietal area | 156168 | 0.01% |
| PEci | Medial parietal cortex | 127008 | 0.01% |
| PIP | posterior intraparietal area | 97632 | 0.01% |

**Supplementary Table S4 Projection signals and proportions of occipital lobe.**

| **Acronym** | **Full name of brain areas** | **Signal Volume (μm³)** | **Signal Ratio** |
| --- | --- | --- | --- |
| V2 | visual area 2 | 32557896 | 2.48% |
| V1 | visual area 1 (primary visual cortex) | 30470256 | 2.32% |
| V4 | visual area 4 (dorsal part) | 18321984 | 1.39% |
| V3v | visual area 3, ventral part | 8127648 | 0.62% |
| V4v | visual area 4, ventral part | 7052616 | 0.54% |
| V3d | visual area 3, dorsal part | 2303640 | 0.18% |
| V3A | visual area V3A | 828144 | 0.06% |
| V2_or_v23b? | visual area 2 | 371088 | 0.03% |
| V6Ad | visual area | 369144 | 0.03% |
| V6 | visual area | 321624 | 0.02% |
| V6Av | visual area | 320328 | 0.02% |
| V2? | visual area 2 | 153144 | 0.01% |
| V4t | visual area 4 | 25056 | 0.00% |

**Supplementary Table S5 Projection signals and proportions of insular regions.**

| **Acronym** | **Full name of brain areas** | **Signal Volume (μm³)** | **Signal Ratio** |
| --- | --- | --- | --- |
| Ial | lateral agranular insula area | 5895936 | 0.45% |
| Iai | intermediate agranular insula area | 4939488 | 0.38% |
| Id | dysgranular insula | 4210920 | 0.32% |
| Ig | granular insula | 1377000 | 0.10% |
| Pi | parainsular area | 1357128 | 0.10% |
| Iam | medial agranular insula area | 625104 | 0.05% |
| Iapm | posteromedial agranular insula area | 340416 | 0.03% |
| Iapl | posterolateral agranular insula area | 293112 | 0.02% |
| G | gustatory cortex | 163512 | 0.01% |
| Ia | agranular insula | 142992 | 0.01% |
| Ri | retroinsula | 56160 | 0.00% |

**Supplementary Table S6 Projection signals and proportions of limbic regions.**

| **Acronym** | **Full name of brain areas** | **Signal Volume (μm³)** | **Signal Ratio** |
| --- | --- | --- | --- |
| 23c | area 23c in posterior cingulate cortex | 5126328 | 0.39% |
| 24c | area 24c in anterior cingulate cortex | 4743576 | 0.36% |
| 24c' | area 24c’ in anterior cingulate cortex | 4513104 | 0.34% |
| 23b | area 23b in posterior cingulate cortex | 2083104 | 0.16% |
| 24b' | area 24b’ in anterior cingulate cortex | 1536408 | 0.12% |
| v23b | area v23b in posterior cingulate cortex | 1130328 | 0.09% |
| 23a | area 23a in posterior cingulate cortex | 824040 | 0.06% |
| 31 | area 31 in the posterior cingulate gyrus | 688176 | 0.05% |
| 30 | area 30 (retrosplenial cortex) | 557280 | 0.04% |
| 24a' | area 24a’ in anterior cingulate cortex | 189864 | 0.01% |
| 29 | area 29 (retrosplenial cortex) | 109080 | 0.01% |
| v23b | visual area 2 | 69552 | 0.01% |

**Supplementary Table S7** **Experimental details of macaque monkeys.**

| ID | Species | Sex | Age | Weight | Injection site | Viral vector | Expression time | | Resolution | Range |
| --- | --- | --- | --- | --- | --- | --- | --- | --- | --- | --- |
| #1 | Macaque fascicularis | M | 8 yr | 3.7 kg | vlPFC | AAV2/9-CaMKIIa-Tau-GFP | | ~90 days | 0.65μm×0.65μm | Right brain |
| #2 | Macaque fascicularis | M | 10 yr | 4.0 kg | vlPFC | AAV2/9-CaMKIIa-Tau-GFP  retroAAV-CaMKIIa-mcherry (retrograde) | | ~120 days  ~120 days | 0.65μm×0.65μm | Whole brain |
| #3 | Macaque fascicularis | M | 9 yr | 3.8 kg | vlPFC | AAV2/9-CaMKIIa-Tau-GFP | | ~75 days | 0.65μm×0.65μm | Right brain |
| #4 | Macaque fascicularis | F | 10 yr | 4.3 kg | vlPFC | AAV2/9-CaMKIIa-Tau-GFP | | ~75 days | 0.65μm×0.65μm | Right brain |

**Abbreviations:** M, male; F, female; vlPFC, ventrolateral prefrontal cortex.

**Supporting Figures**


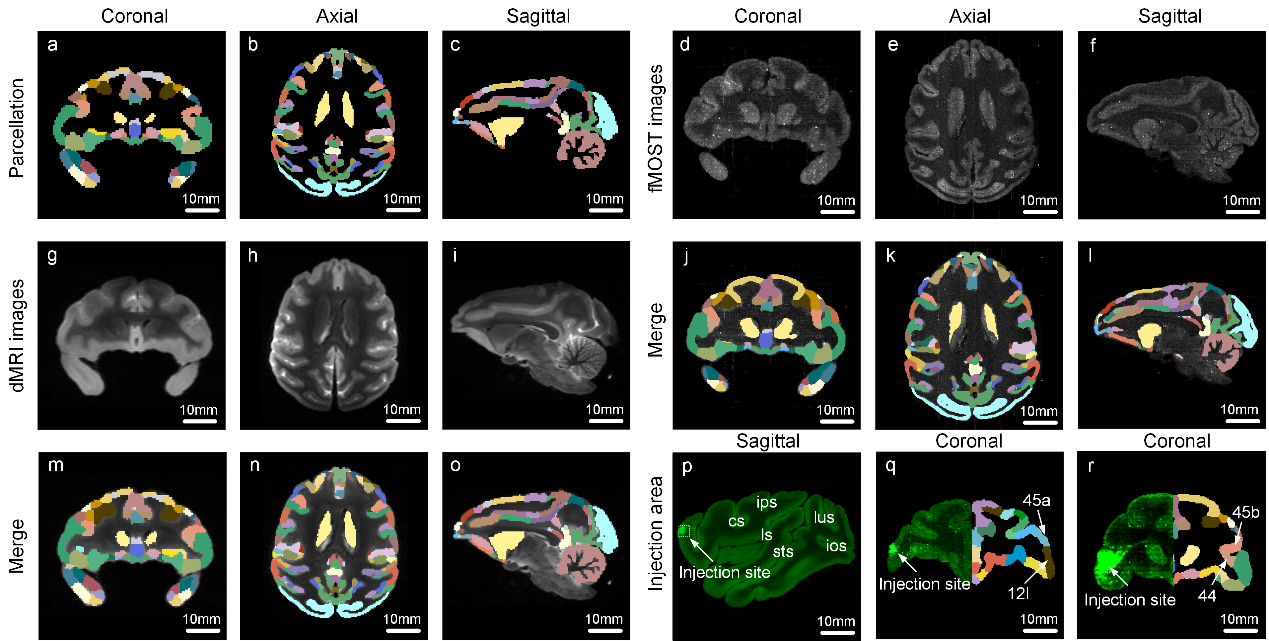


**Figure S1: (a-c)** Coronal, horizontal, and sagittal views of the D99 atlas utilized for cortical parcellation at a resolution of 500 × 500 × 500 μm). **(d-f)** Coronal, horizontal, and sagittal views of fMOST images, shown at a resolution of 300 × 300 × 300 μm^3^). **(g-i)** Coronal, horizontal, and sagittal views of dMRI images acquired at a resolution of 500 × 500 × 500 μm). **(j-l)** Registration of the atlas and fMOST images to a common space, presented in coronal, horizontal, and sagittal planes. **(m-o)** Registration of the atlas and dMRI images to a common space, presented in coronal, horizontal, and sagittal planes. **(p-r)** Representative sagittal and coronal slices of the GFP channel, showing the injection site in the vlPFC overlaid with the D99 macaque atlas, primarily encompassing areas 44, 45a, 45b, and 12l.


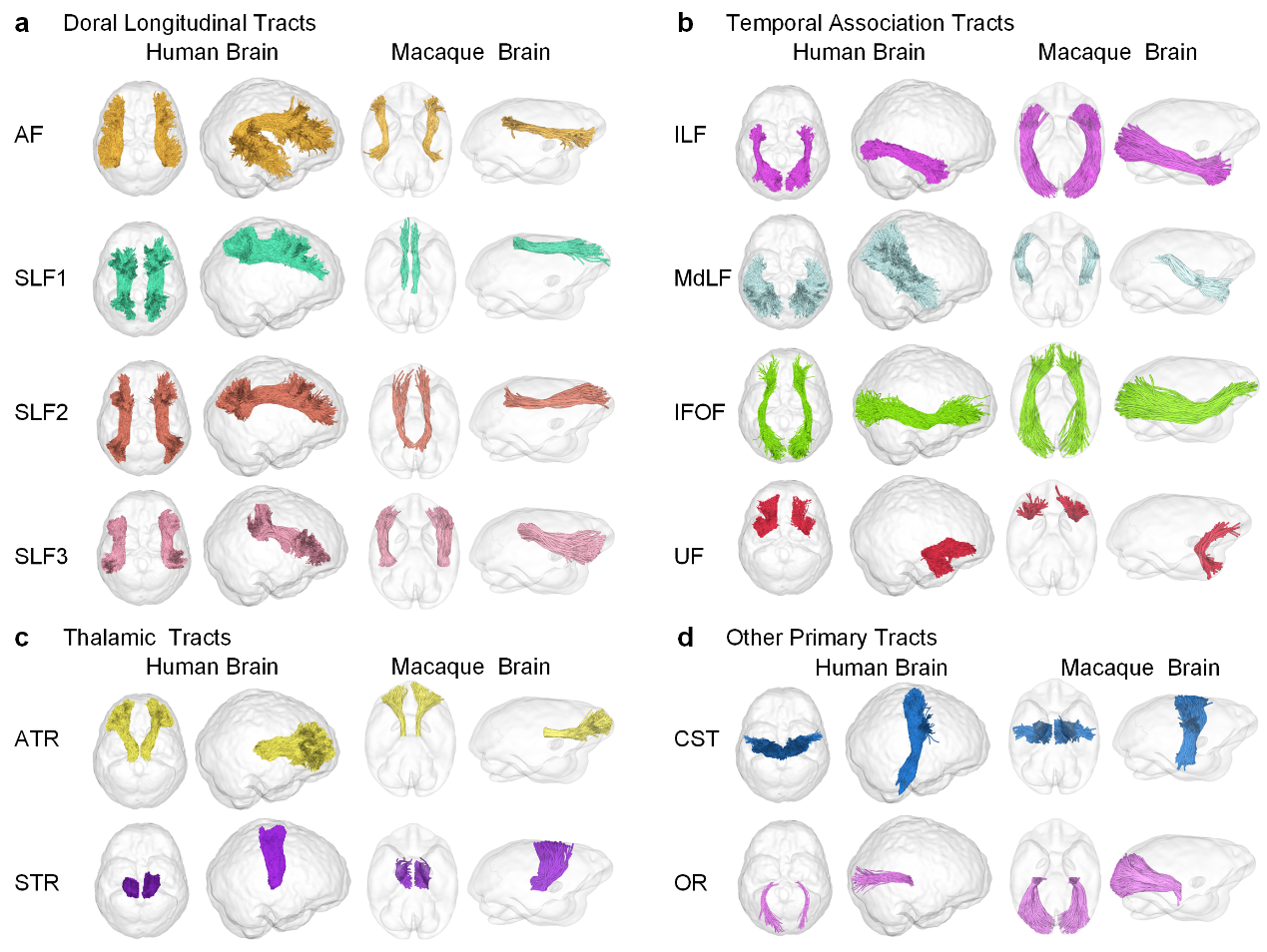


**Figure S2 The tractography results for both human and macaque brains were classified into four distinct group. (a)** Dorsal Longitudinal Tracts: This group includes the arcuate fasciculus (AF), superior longitudinal fasciculus 1 (SLF1), superior longitudinal fasciculus 2 (SLF2), and superior longitudinal fasciculus 3 (SLF3). **(b)** Temporal Association Tracts: This group encompasses the inferior longitudinal fasciculus (ILF), middle longitudinal fasciculus (MdLF), fronto-occipital fasciculus (IFOF), and uncinate fasciculus (UF). **(c)** Thalamic Tracts: This category comprises the anterior thalamic radiation (ATR) and superior thalamic radiation (STR). **(d)** Other Primary Tracts: This group includes the corticospinal tract (CST) and optic radiation (OR). The tracts are presented from both inferior and lateral perspectives.

**
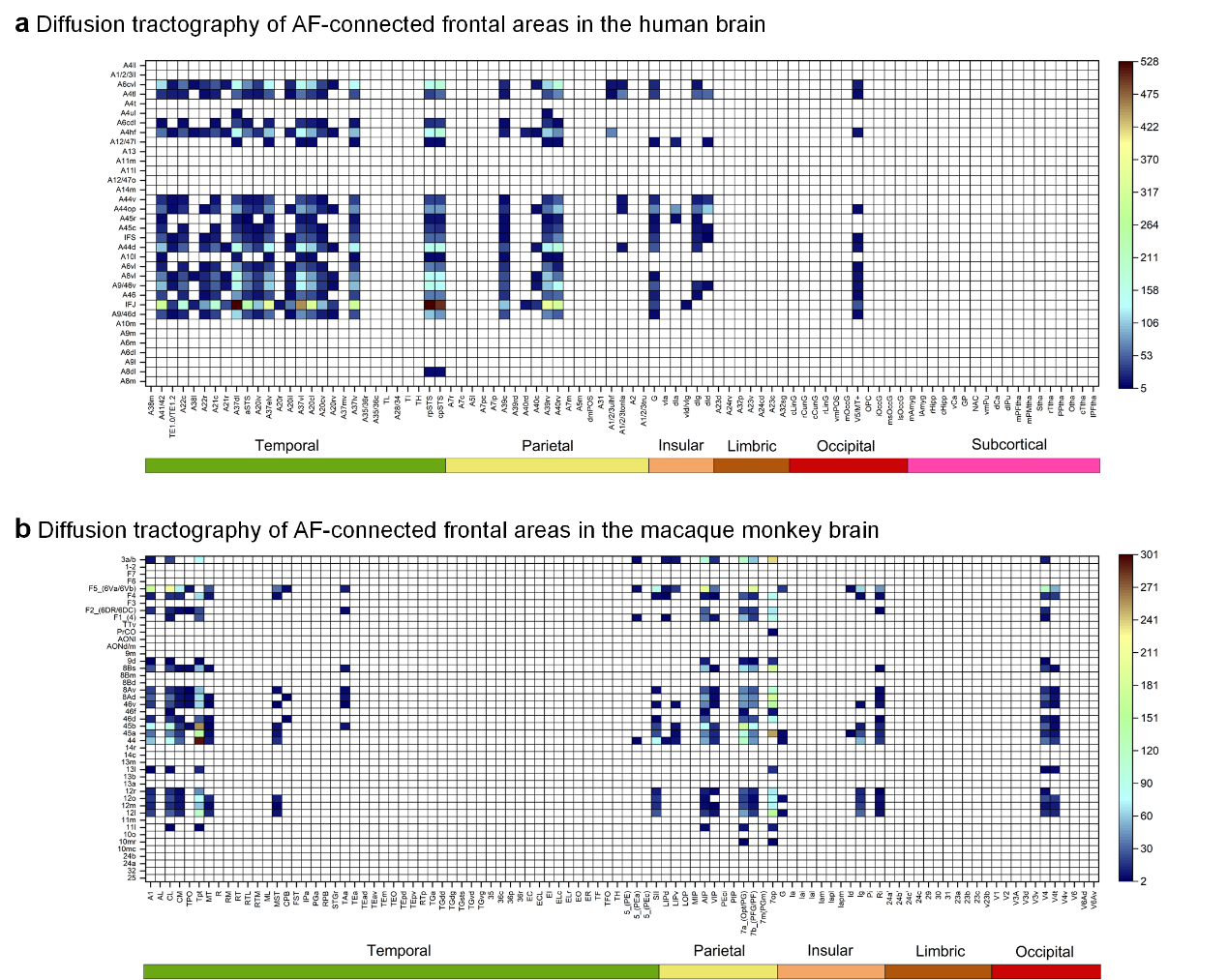
**

**Figure S3 Connections between the AF-connected frontal areas and other cortical areas in two species brains as shown by diffusion tractography. (a)** Connectivity map shows the structural connectivity of the AF-connected frontal areas to cortical areas in the human brain. Connection measured by the number of fibers is displayed using the color bar on the right. The number of streamlines shown was thresholded at 5 and values less than this are shown as blank. **(b)** Connectivity map shows the structural connectivity of the AF-connected frontal areas to cortical areas in the macaque brain. The number of streamlines shown was thresholded at 5 and values less than this are shown as blank. The colored labeled bars at the bottom indicate cortical divisions, with parcellation based on the Brainnetome and D99 atlas


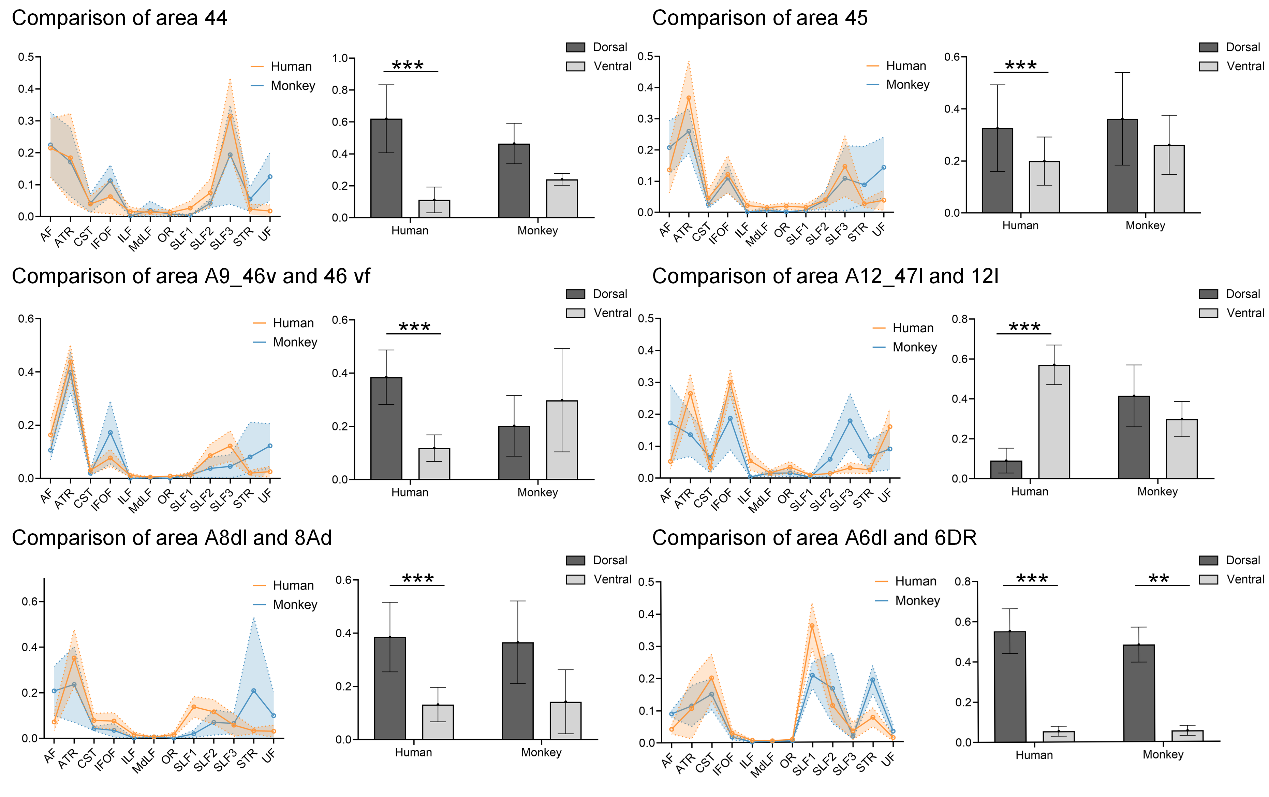


**Figure S4 Comparison of connectivity profiles of dorsal and ventral pathways in homologous regions within the frontal lobe.** Areas 44 and 45, A9_46v in humans and area 46vf in macaques; A12_47l in humans and 12l in macaques; A8dl in humans and 8Ad in macaques; A6dl in humans and area 6DR in macaques. Each subplot contains a line graph representing the average probability distribution of different tracts within each homologous region, with the shaded area indicating the range of standard deviation (STD). Bar charts accompany these graphs, illustrating the average probability results for the ventral (including IFOF, ILF, MdLF, and UF) and dorsal pathways (including AF, SLF1, SLF2, and SLF3) across the two species. The significance levels are denoted as follows: ns ≥ 0.05; 0.05 > * ≥ 0.01; 0.01 > ** ≥ 0.001; 0.001 > ***. The analysis revealed that in area 44, the tracts AF and SLF3 are predominant in both humans (AF: average = 0.213, STD = 0.068; SLF3: average = 0.305, STD = 0.153) and macaques (AF: average = 0.225, STD = 0.102; SLF3: average = 0.194, STD = 0.134). Furthermore, the comparison between ventral and dorsal pathways showed that in humans, the probability of dorsal pathway fibers is significantly higher than that of ventral pathway fibers in areas 44, 45, A9_46v, and A8dl. In contrast, no significant differences were observed in these regions in the macaque brain. Additionally, in the human A12_47l region, the ventral pathway (average = 0.57, STD = 0.10) is significantly more pronounced than the dorsal pathway (average = 0.09, STD = 0.06) as demonstrated by the Mann-Whitney U Test (p = 6.80 × 10^-8^).


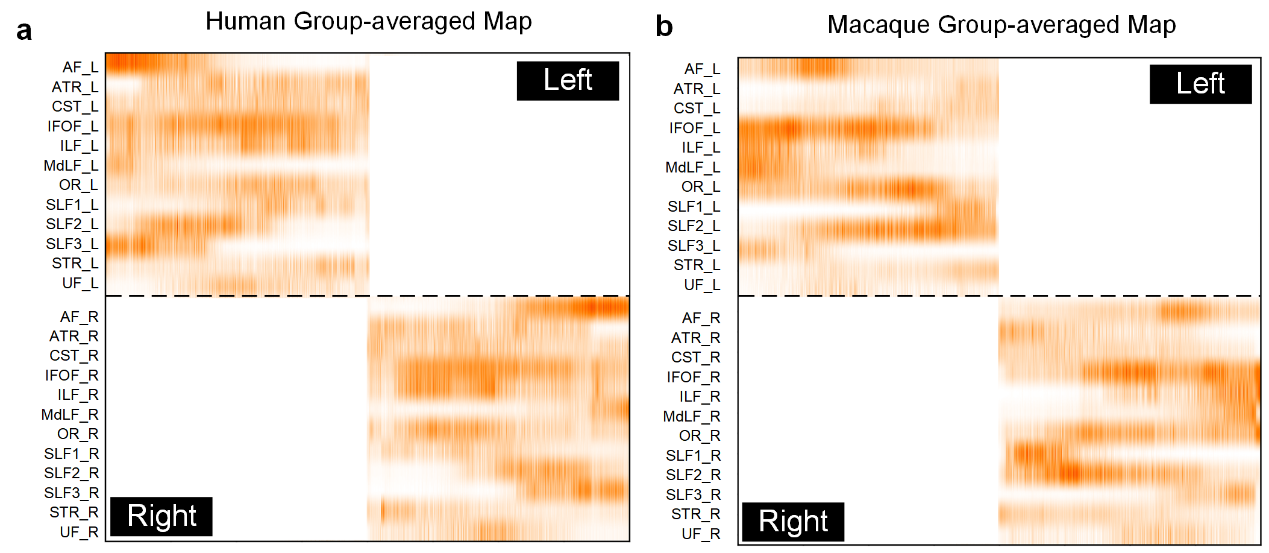


**Figure S5 Group-averaged connectivity blueprints of humans and macaques.** In these blueprints, the x-axis represents 20,002 distinct points on the cortical surface, while the y-axis corresponds to 12 homologous tracts in both human and macaque brains. Each point in the figure indicates the probability of the corresponding tract traversing a specific cortical region. For each vertex, the sum of the probabilities across all tract connections is normalized to 1.

**
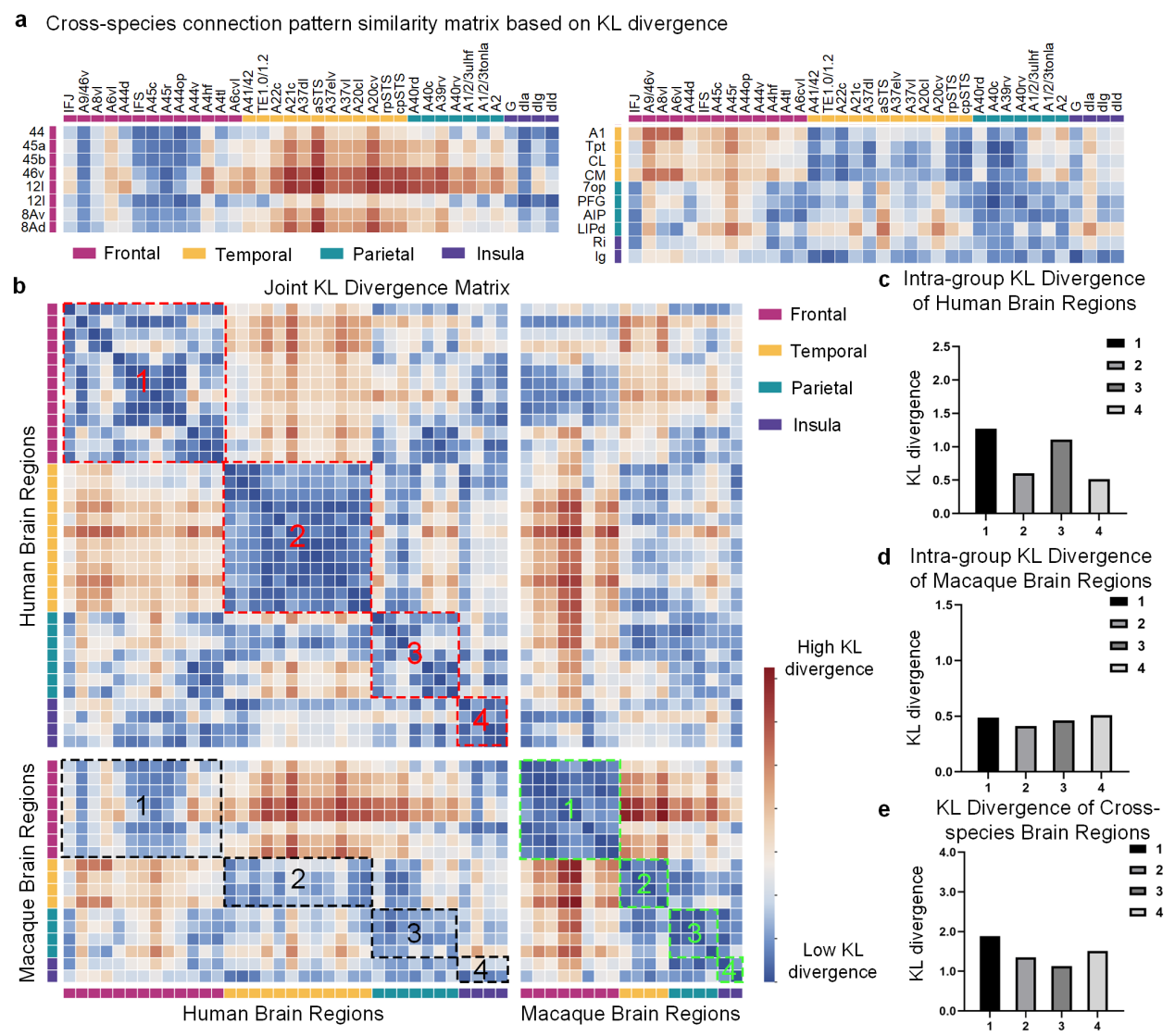
**

**Figure S6 The KL divergence matrix. (a)** The KL divergence matrix compares AF-relevant regions between human and macaque brains. The x-axis represents cortical regions in the human brain, while the y-axis represents corresponding regions in the macaque brain. The frontal, temporal, parietal, and insular cortex regions are color-coded as red, yellow, cyan, and purple, respectively. **(b)** The joint KL divergence matrix aggregates all AF-relevant cortical regions across both human and macaque brains. c-e. The average intra-group KL divergence values are presented as follows: **(c)** Within human brain areas (1: frontal lobe, 2: temporal lobe, 3: parietal lobe, 4: insular lobe, indicated by red dashed boxes in **(b)**). **(d)** Within macaque cortical regions (indicated by green dashed boxes in **(b)**). **(e)** Between human and macaque cortical regions (indicated by black dashed boxes in **(b)**). The analysis reveals that the maximum average KL divergence is observed in the cross-species comparisons of the frontal regions (1.89, STD = 1.19), whereas the minimum value (0.54, STD = 0.23) occurs in the temporal lobe comparisons within macaque cortical regions.


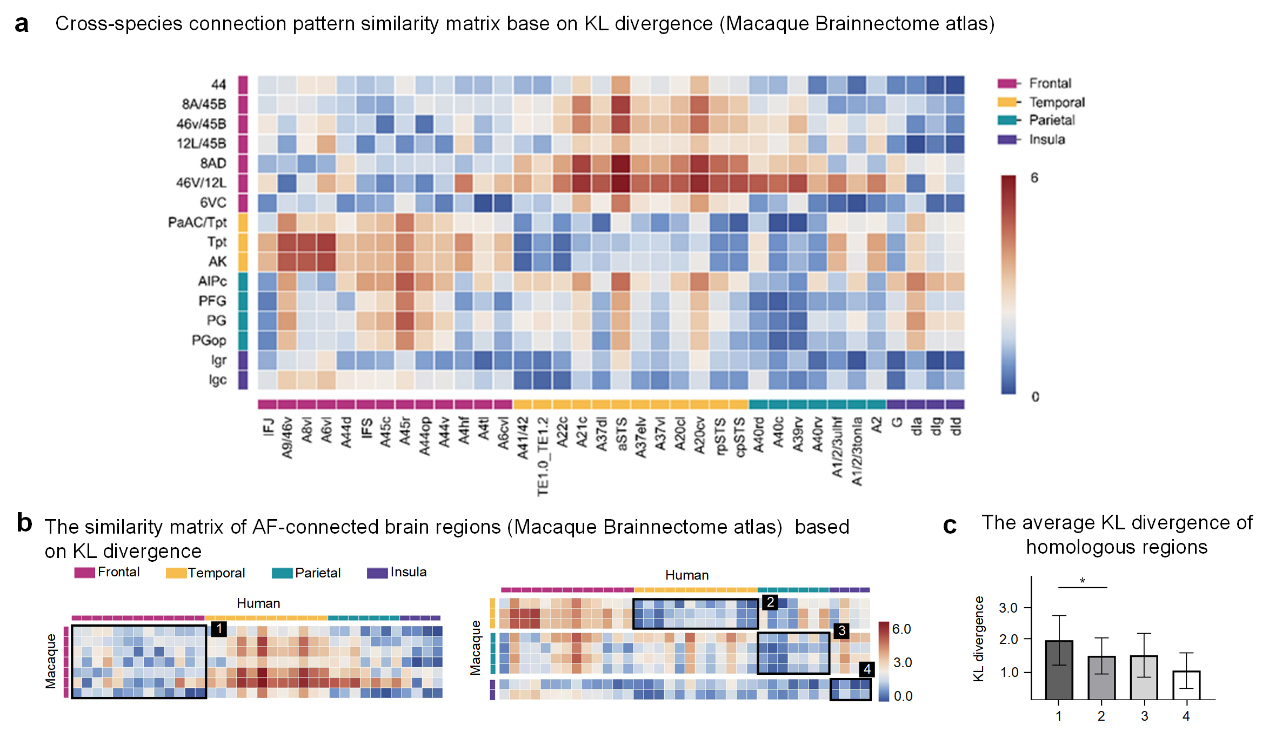


**Figure S7 The KL divergence matrix based on Macaque Brainnectome atlas.** (a) & (b) The KL divergence matrix aggregates all AF-relevant cortical regions across both human and macaque brains. Vertical labels correspond to macaque brain parcels, and horizontal labels correspond to human brain parcels. (c) The mean KL divergence among regions within the same lobes is indicated by the black box in (b): 1 for frontal, 2 for temporal, 3 for parietal, and 4 for insular regions. Results based on the Macaque Brainnectome atlas showed that the average KL divergence in the frontal lobe (2.03 ± 2.83) was significantly higher than that in the temporal lobe (1.39 ± 1.15, FDR-corrected p < 0.05), exhibiting a trend consistent with results based on the D99 atlas (**Figure 6c-d**). Further analysis of the Broca's homolog area and area Tpt (regions of interest) revealed that the macaque Broca's homolog area and the human frontal lobe area (average KL divergence of areas: 44, 2.07 ± 0.47; 8A/45B, 2.02 ± 0.36; 46v/45B, 1.98 ± 0.74; 12L/45B, 1.98 ± 0.95) exhibit greater dissimilarity. It is worth noting that macaque area Tpt (1.38 ± 0.48)—the shared region of AF projections in the temporal lobe—shows consistency in connection patterns. Results indicate that the consistency of species differences in AF connection patterns within the projection regions—revealed by cross-species analysis—is not affected by brain parcels.
